# Raffinose degradation-related gene *GhAGAL3* was screened out responding to salinity stress through expression patterns of *GhAGALs* family genes

**DOI:** 10.3389/fpls.2023.1246677

**Published:** 2023-12-19

**Authors:** Wenhua Chen, Yupeng Cui, Yunxin He, Lanjie Zhao, Ruifeng Cui, Xiaoyu Liu, Hui Huang, Yuexin Zhang, Yapeng Fan, Xixian Feng, Kesong Ni, Tiantian Jiang, Mingge Han, Yuqian Lei, Mengyue Liu, Yuan Meng, Xiugui Chen, Xuke Lu, Delong Wang, Junjuan Wang, Shuai Wang, Lixue Guo, Quanjia Chen, Wuwei Ye

**Affiliations:** ^1^ Institute of Cotton Research of Chinese Academy of Agricultural Sciences/Research Base, Anyang Institute of Technology, National Key Laboratory of Cotton Bio-breeding and Integrated Utilization, Anyang, Henan, China; ^2^ Engineering Research Centre of Cotton, Ministry of Education/College of Agriculture, Xinjiang Agricultural University, Urumqi, China; ^3^ Hunan Institute of Cotton Science, Changde, Hunan, China

**Keywords:** α-galactosidase (AGALs), *Gossypium hirsutum*, raffinose family oligosaccharides (RFOs), abiotic stresses, functional verification

## Abstract

A-galactosidases (AGALs), the oligosaccharide (RFO) catabolic genes of the raffinose family, play crucial roles in plant growth and development and in adversity stress. They can break down the non-reducing terminal galactose residues of glycolipids and sugar chains. In this study, the whole genome of AGALs was analyzed. Bioinformatics analysis was conducted to analyze members of the AGAL family in *Gossypium hirsutum*, *Gossypium arboreum*, *Gossypium barbadense*, and *Gossypium raimondii.* Meanwhile, RT-qPCR was carried out to analyze the expression patterns of AGAL family members in different tissues of terrestrial cotton. It was found that a series of environmental factors stimulated the expression of the *GhAGAL3* gene. The function of *GhAGAL3* was verified through virus-induced gene silencing (VIGS). As a result, *GhAGAL3* gene silencing resulted in milder wilting of seedlings than the controls, and a significant increase in the raffinose content in cotton, indicating that *GhAGAL3* responded to NaCl stress. The increase in raffinose content improved the tolerance of cotton. Findings in this study lay an important foundation for further research on the role of the *GhAGAL3* gene family in the molecular mechanism of abiotic stress resistance in cotton.

## 1 Introduction

Plants are subjected to a variety of abiotic stresses in their growth process, including drought, salt, high temperature, and cold stresses. To enable the normal growth of plants under various stress conditions, plants have developed multiple signaling and regulatory pathways to withstand stress and thrive. Raffinose family oligosaccharides (RFOs), the essential substances mediating stress response in plants, play an important role in enhancing plant resistance to abiotic stresses (Li et al., 2011). Studies have reported that the expression of genes related to RFO metabolites is significantly upregulated under stress conditions such as low temperature, drought, and high salt. These findings suggest that RFOs may have a crucial effect on enhancing resistance to abiotic stresses (Joshi et al., 2021; Lee et al., 2021). In addition, studies have reported that RFOs can facilitate the elimination of reactive oxygen species (ROS) in plants experiencing stress (Ma et al., 2021). Raffinose can be transported to the chloroplast, protects the thylakoid, and maintains the stability of the photosynthetic system, PSII (Schneider and Keller, 2009). As ROS scavengers, both raffinose and galactose can trap hydroxyl radicals and reduce oxidative damage in plants under stressful conditions (Nishizawa-Yokoi et al., 2008). AGAL, the gene that breaks down RFOs in plants, has received little attention, but it is just as important as the gene that synthesizes RFOs.

AGAL is a ubiquitous enzyme in the plant kingdom (Wang et al., 2022). AGALs of Family 26 are of eukaryotic origin, whereas the AGALs of Family 37 is mainly of prokaryotic origin (Fialho Lda et al., 2008). A notable function of this enzyme is in the germination of seeds and tubers (Hughes et al., 2016). AGAL exhibits a high activity during seed maturation, during germination, and in the seedling stage, which can be attributed to hydration factors that result in the complete degradation of soluble sugars in the embryo axis and primarily in the cotyledons (Mittal and Sharma, 1991). AGAL is involved in numerous aspects of plant metabolism, including the hydrolysis of α-1,6 chains of cotton seed oligosaccharides during deforming. Downregulation of *α-Gal* gene expression in Petunia spp. results in increased whole plant frost resistance in non-domesticated and cold-domesticated plants. In contrast, overexpression of the *α-Gal* gene leads to the reduced endogenous raffinose and decreased frost resistance (Pennycooke et al., 2003). *Osh69*, a rice alkaline galactosidase, was found to be located in chloroplasts and to play a role in leaf senescence. It is upregulated in darkness and in response to injury (Lee et al., 2004). Meanwhile, the *AGA3* protein was identified in cucumber chloroplasts and its expression was downregulated under cold stress. However, it was upregulated after temperature recovery. This strongly suggests that *AGA3* plays an important role in the catabolism of chloroplast RFOs under cold stress. Notably, the alkaline environment of the chloroplast stroma is suitable for *AGA3* to exert its catalytic action. *AGA2*, another alkaline galactosidase, is responsible for the catabolism of RFOs in the cytoplasm once the temperature returns to the normal level. RFOs accumulate in different subcellular compartments of cucumber leaves under cold stress, whereas upon the removal of the stress, they can be broken down *in situ* by various galactosidases (Sui et al., 2012; Gu et al., 2018). In the research on spinach in New Zealand, the expression of the *TtAGAl1* gene was affected by various abiotic stresses. Among them, drought was found to be a particularly strong promoter of *TtAGAl* expression (Hara et al., 2008). Moreover, the expression of related genes in *Vitis vinifera* was studied under salt and drought stresses as well as and during seed development. It was found that the *Vv-a-gal/SIP* gene is differentially expressed under different osmotic stress conditions. Additionally, *Vv-α-gal/SIP*-specific transcripts were preferentially accumulated in salt-tolerant *Vitis vinifera* varieties (Daldoul et al., 2012). *ZmAGA1* expression was enhanced in maize seedlings under cold and drought stress conditions, but not upon sodium chloride stress (Zhao et al., 2006). *AGAL* is an essential enzyme for plant growth and development, which plays a crucial role in plant development Although characterized *AGAL* has been studied in several species, it has not been reported in cotton. It is crucial to delve into the mechanisms underlying the salinity stress resistance in cotton.

Cotton is an economically important crop that is grown worldwide for the production of fiber and cottonseed oil. However, stress conditions often affect the growth and development of cotton, thereby reducing its yield. Different abiotic stresses may have diverse effects on cotton (Bawa et al., 2022). With the rapid development of sequencing technology, the genome sequences of *Gossypium hirsutum*, *Gossypium arboreum*, *Gossypium barbadense*, and *Gossypium raimondii* have been sequenced and resolved. In this study, the *AGAL* family of cotton was identified and characterized by bioinformatics analyses (Huang et al., 2022).The expression pattern of the *GhAGALs* gene was also analyzed, and the results showed that *GhAGALs* expression varied in different tissues. In addition, the *AGAL* gene plays a role in the function of *Gossypium hirsutum* and its specific site of action. Findings in this study provide a theoretical foundation for future studies on the utilization of the *AGAL* family in *Gossypium hirsutum.*

## 2 Experimental materials and methods

### 2.1 Identification of AGAL family members and construction of evolutionary tree

The protein sequences of four major cotton species *Gossypium hirsutum*, *Gossypium arboreum*, *Gossypium barbadense*, and *Gossypium raimondii* were downloaded from the cotton database Cotton FGD (https://cottonfgd.org), *Gossypium hirsutum* (ZJU), *Gossypium arboreum* (CRI), *Gossypium barbadense* (ZJU), *Gossypium raimondii* (JGI), the genome sequence, and the CDS sequence (Zhu et al., 2017). Additionally, protein sequences, genomes, and other biological information of five diploid species were downloaded using the online database JGI Phytozome v12.1. *Arabidopsis thaliana*, *Oryza sativa*, *Populus trichocarpa*, *Vitis vinifera*, and *Zea mays* (Wang et al., 2021). Furthermore, the hidden Markov model (HMM) file for the conserved domain (PF16499) was downloaded from the Pfam database (https://pfam.xfam.org/). Hidden Markov models were used with (PF16499) as the query file to search for candidate *AGAL* family genes in the genomes of cotton and other species. This search was conducted using HMMER (version 3.3.1) (http://www.hmmer.org/) and BLASTP. Manual removal of redundant sequences from HMMER (version 3.3.1) and BLASTP results for incomplete genes (Henderson et al., 1997). These genes were identified as members of the *AGAL* gene family. The identified *AGAL* family members were renamed *GhAGAL1-GhAGAL15* based on their chromosomal positions (Zhang et al., 2021).

To clearly understand the evolutionary relationship of the *AGAL* gene, those identified *AGAL* gene family members were used to search for protein sequences of cotton and five diploid species: *Arabidopsis thaliana*, *Oryza sativa*, *Populus trichocarpa*, *Vitis vinifera*, and *Zea mays.* We employed using HMMER (version 3.3.1) and BLASTP for this purpose. Multiple sequence alignment was performed using MEGA 7.0 software (Cui et al., 2021b). Construction of intraspecific and interspecific evolutionary trees for *AGAL* using the online software ChiPlot (Xie et al., 2023) online tools to enhance the visualization of evolutionary trees (Kumar et al., 2016).

### 2.2 Chromosome localization in the *AGAL* family

Biological information concerning the location and structure of *AGAL* family members was extracted from the genome gff3 annotation files of four cotton species (Chen et al., 2018). Afterward, locations of the cotton *AGAL* family genes on the chromosome were analyzed and mapped with TBtools software (Chen et al., 2020; Cai et al., 2021), so as to reveal the coevolutionary relationships between the *AGAL* families of cotton species.

### 2.3 Gene structure and conserved motif analysis

Phylogenetic tree, gene structure, and conserved protein motif were triply mapped with MAST files, GhAGALs evolutionary tree NWK files, and gff3 files by adopting TBtools software.

### 2.4 Analysis of *GhAGALs* gene promoter elements and expression level analysis

To investigate the relationships of the *AGAL* family with hormones and stress, the upstream 2,000-bp sequence (Malik et al., 2020) of the start codon in the *AGAL* family was selected from the *Gossypium hirsutum* genome sequence by using the PlantCARE data database query system (http://bioinformatics.psb.ugent.be/webtools/plantcare/html/) (Lescot et al., 2002). Afterward, *cis*-acting elements in the upstream regions of genes were identified, analyzed and later visualized with TBtools after screening (Chen et al., 2020).

### 2.5 Analysis of the tissue expression pattern of *GhAGALs* family genes

To analyze the expression of *GhAGALs* in various tissues, *GhAGALs* data were obtained from the online database of the Cotton Research Institute (http://grand.cricaas.com.cn/page/tools/expressionVisualization). In addition, representative organization values for root, stem, and leaf tissues were selected based on the characteristics of the *GhAGALs* family. The original data were analyzed to obtain the gene expression levels, which were subsequently visualized using a histogram.

### 2.6 Gene interaction network

The GhAGAL protein interaction network was analyzed based on the STRING database (https://string-db.org/) after taking *Arabidopsis thaliana* orthologs into consideration, so as to predict the interactions between GhAGAL family genes and other genes in cotton.

### 2.7 RNA isolation and quantitative reverse transcription-polymerase chain reaction of *GhAGALs* family genes

*Gossypium hirsutum* plants, provided by the Institute of Cotton Research of the Chinese Academy of Agricultural Sciences (Zhong 9807), were grown in an incubator at 25°C.When the cotton grew to three leaves and one heart stage, the plants were subjected to the stress of 100 mM NaCl, and the leaves were collected at 0 h, 6 h, 12 h, and 24 h. RNA was then extracted using the Aidlab kit. cDNA was synthesized through reverse transcription using TransGen Biotech. Clear water-treated plants were used as the control. The procedure was as follows: 94°C for 30 s; 94°C for 5 s, 55°C for 15 s, and 72°C for 10 s for 45 cycles; followed by storage at 4°C (**Supplementary Table S1**). The relative expression of genes was calculated using the 2^−ΔΔCt^ method, with actin being the internal reference (Chen et al., 2020).

### 2.8 Construction of the *GhAGAL3* recombinant vector and transformation by *Agrobacterium tumefaciens*

Firstly, we downloaded the CDS sequence of *GhAGAL3* from the Cotton FGD database. Then, a 300-bp region was selected from the silent site to design VIGS primers for amplifying the target fragment. The pYL156 silencing vector was constructed by double digesting the pYL156 vector with two restriction enzymes, *BamHI* and *SacI*, using the In-Fusion technique. Next, the recombinant vector product was transfected into *E. coli*, PCR was performed using VIGS primers, and the resulting bands were sent to the testing company for sequencing. After obtaining the correct sequencing results, re-transformation with *Agrobacterium tumefaciens* was conducted. In brief, the LBA4404 bacteriophage carrying the following constructs, including control pYL156 (empty vector), pYL156: *GhAGAL3*, pYL156: PDS (positive control), and pYL192 (help vector), was inserted into the cotyledons of Zhong 9807. After 24 h of dark treatment, cotton was grown in an incubator at 25°C/16-h light and 23°C/8-h dark cycle conditions (Fan et al., 2022). When cotton grew to three leaves and one heart stage, lines injected with pYL156 and successfully silenced were immersed in the 100-mM NaCl solution, whereas control lines were immersed in ddH_2_O. After 36 h, phenotypic differences were observed among the various treatments and individual plants were sampled. Each sample weighed 0.1 g.

### 2.9 Determination of raffinose, D-galactose, and D-glucose contents

The leaves of control and NaCl-treated cotton seedlings were collected at each time point, immediately frozen in liquid nitrogen, and stored at −80°C to determine the raffinose content. The content of raffinose was detected using a plant raffinose ELISA kit, that of D-galactose content using the D-galactose content kit (Article number: ADS-W-TDX046). D-Glucose content test was performed using the D-glucose content (GOPOD oxidase method) kit (Article number: ADS-W-TDX002).

### 2.10 Determination of Pro and MDA contents

For determination of proline (Pro) and malondialdehyde (MDA) contents, the leaves of control and NaCl-treated cotton seedlings were collected at each time point and immediately frozen in liquid nitrogen and stored at −80°C to determine the content of Pro and MDA. The content of Pro was determined using the test kit from Nanjing Jiancheng Bioengineering Institute, the content of MDA was determined using the test kit from Beijing Solarbio Science & Technology Co., Ltd. (Liu et al., 2022; Meng et al., 2023).

### 2.11 Determination of chlorophyll content

Chlorophyll was extracted from 0.1 g of fresh cotyledon leaves by overnight immersion in an 85% (v/v) acetone solution, the supernatant was centrifuged at 4,000 rpm for 10 min and diluted with 85% acetone to the appropriate concentration, and absorbance values were measured by absorbance at 424.5, nm 644 nm, and 663 nm, with 85% acetone serving as a blank (Farooq et al., 2013).

### 2.12 Leaf DAB staining

First, the DAB working solution was made in accordance with the DAB stain kit’s instructions and kept chilled at 4°C. The leaves were removed from the staining solution and soaked in anhydrous ethanol. The ethanol can be changed several times during the decolorization process to ensure that the green color of the leaves disappears. Cotton samples were submerged in the staining solution and stained overnight in the dark. When the green tint had finally faded completely, ROS distribution and accumulation was seen.

### 2.13 Statistical analysis

A minimum of three biological replicates was deemed necessary for each dataset. Spass19.0 was used for histogram drawing, and GraphPad Prism 9.0 was used for data significance analysis.

## 3 Results

### 3.1 Identification of *AGAL* family members and construction of the evolutionary trees

**Figure 1:**
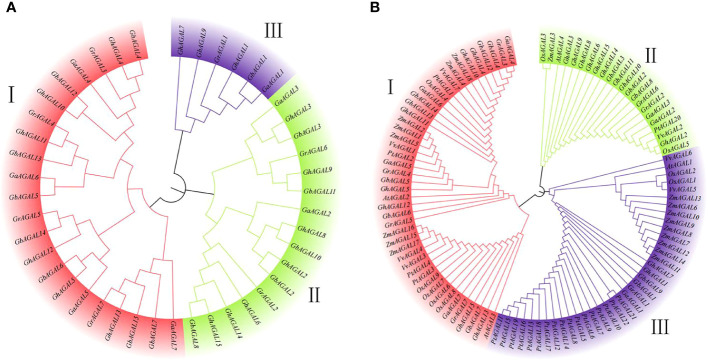
Phylogenetic tree constructed with MEGA7 by the neighbor-joining (NJ) method. **(A)** Evolutionary analysis of the *AGAL* family members of four cotton species, **(B)** Phylogenetic relationships of *AGAL* genes from four cotton species and five other plant species.

To further understand the evolutionary relationships of the *AGAL* family in cotton, nine different species were identified in this study, namely, *Gossypium hirsutum*, *Gossypium arboreum*, *Gossypium barbadense*, *Gossypium raimondii*, *Oryza sativa*, *Arabidopsis thaliana*, *Zea mays*, *Populus trichocarpa*, and *Vitis vinifera*. According to our identification results, 15 genes were in *Gossypium hirsutum*, 15 in *Gossypium barbadense*, 7 in *Gossypium raimondii*, 7 in *Gossypium arboreum*, 9 in *Oryza sativa*, 7 in *Vitis vinifera*, 18 in *Zea mays*, 22 in *Populus trichocarpa*, and 4 in *Arabidopsis thaliana*. Using MEGA 7.0 software, the amino acid sequences were compared and analyzed by the maximum likelihood method, and a phylogenetic tree was constructed (**Figure 1A**). The classification of the genes within the species’ family can be clearly observed through the phylogenetic tree. The genes are divided into three subclasses: I, II, and III. Subclass I was further divided into two subfamilies, and subclass II and subclass III were also divided into two subfamilies. The (**Figure 1B**) shows that the genes of subclass I and subclass III families tend to converge, whereas subclass II had the lowest number of family genes. The closest relationship was seen between cotton and *Zea mays*. Thereafter, the evolutionary trees were compared, which showed that the number of genes in *Gossypium hirsutum* and *Gossypium barbadense* was higher than that in *Vitis vinifera*, *Oryza sativa*, and *Arabidopsis thaliana* (Rui et al., 2022). This suggested that cotton underwent massive amplification during the evolutionary process (**Figure 1B**).

To further analyze the evolutionary relationships among the common ancestors of the four cotton species, their amino acid sequences were compared and analyzed with MEGA 7.0 software. In addition, the neighbor-joining method was applied in constructing the phylogenetic tree of the *AGAL* family (**Figure 1B**). As clearly observed from the phylogenetic tree, the species’ family genes were classified into three subclasses I, II, and III. Among the homologous genes of *Gossypium hirsutum*, the *AGAL* gene was subjected to stable selection across different cotton species. Meanwhile, there were only a few genes undergoing natural selection, as indicated by the conserved amino acid phenotype of the protein.

### 3.2 Chromosome distribution of the *AGAL* family genes in cotton

**Figure 2:**
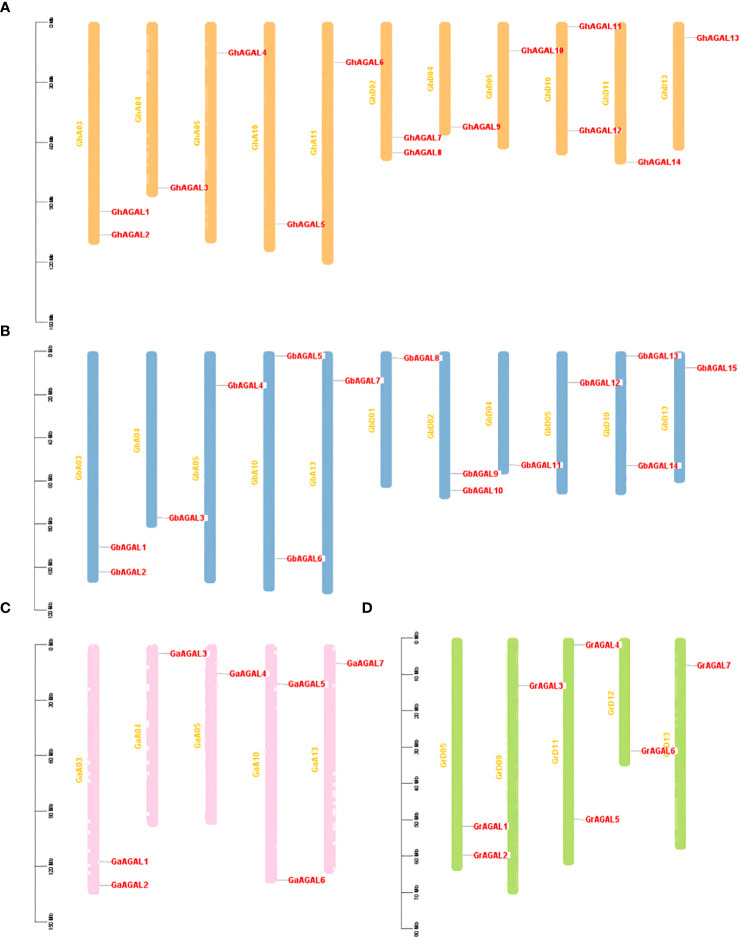
Chromosomal localization of *AGAL* Chromosome distribution of *AGAL* genes in four cotton species. **(A)** The chromosomes of *Gossypium hirsutum*, **(B)** The chromosomes of *Gossypium barbadense*. **(C)** The chromosomes of *Gossypium arboreum*. **(D)** The chromosomes of *Gossypium raimondii*.

We further investigated the distribution of *AGAL* family genes on chromosomes. It was found that the genes were unevenly distributed among certain chromosomes or closely aligned regions (**Figure 2**). Among them, 14 *GhAGALs* family genes of cotton were located on 11 chromosomes and distributed on each of these chromosomes. However, *GhAGAL15* did not appear in chromosome localization, possibly indicating that it was not located on any specific chromosome. Moreover, the 15 *GbAGALs* family genes in *Gossypium barbadense* were also localized on 11 chromosomes, with distribution on each of these chromosomes. The localization of the *AGAL* family genes on chromosomes was generally similar in both *Gossypium barbadense* and *Gossypium hirsutum*, suggesting a certain degree of similarity between these different species. Additionally, all the seven genes of evolutionary *Gossypium arboreum* and *Gossypium raimondii* were evenly distributed on each chromosome. Upon further analysis, most of the genes were highly conserved in their location on the chromosomes. For example, the positions of *GhAGAL1-GbAGAL1-GaAGAL1-GrAGAL1* and *GhAGAL2-GbAGAL2-GaAGAL2-GrAGAL2* on the chromosomes all show similar locations of gene distribution (**Supplementary Table S2**).

### 3.3 Conserved protein motifs and gene structure analysis

**Figure 3:**
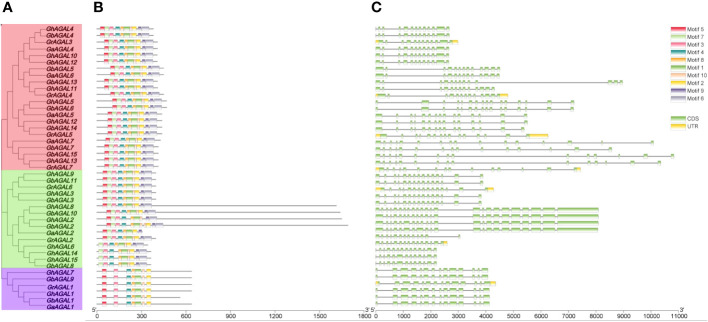
Conserved motifs and gene structure analysis of the GhAGAL family. **(A)** Phylogenetic tree of the GhAGAL family. **(B)** Conserved motifs of the GhAGAL family. **(C)** Gene structure of the GhAGAL family gene structure.

Triadic joint analysis of four major cotton species was conducted by phylogenetic tree, gene structure, and conserved motifs (**Figure 3**). From the figure, it was seen that family members on the same evolutionary branch displayed a similar distribution of conserved motif, which suggested that the family members were functionally similar and structurally evolutionarily conserved (Bailey and Elkan, 1994).

### 3.4 Analysis of cis-acting elements and expression levels in *Gossypium hirsutum*

**Figure 4:**
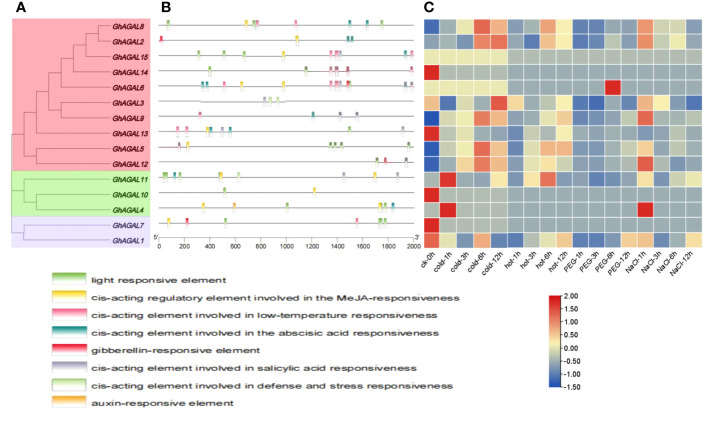
Analysis of *cis*-acting elements and differential expression patterns of the *GhAGAL* gene family members. **(A)** Phylogenetic tree of the *GhAGAL* gene family. **(B)** *cis*-Acting elements in the promoters of the *GhAGAL* gene family members. **(C)** Differential expression levels of *GhAGAL* gene family members in response to cold, heat, salt, and PEG stresses.

To further investigate the mechanisms of the response of *GhAGAL* gene family members to abiotic stresses, relevant data were downloaded to analyze the differential expression of genes under cold, heat, salt, and PEG stresses. As observed from **Figure 3**, the expression of different genes changed to varying degrees under different stresses. Therefore, it was speculated that the *GhAGAL* gene family was involved in the regulation of abiotic stresses (**Figure 3**). In addition, members of the *GhAGAL* gene family also played a crucial role in the physiological and biochemical processes of plants. According to **Figure 4**, the *GhAGAL* gene family is involved in various environmental stimuli, including light response, methyl jasmone, low-temperature stress, abscisic acid, gibberellin, defense, and stress response (Hu et al., 2019) (**Supplementary Table S3**). Therefore, it inferred that the *Gossypium hirsutum GhAGALs* family primarily consists of phytohormones and *cis*-acting elements related to adversity. Afterward, the expression of 15 genes in the *AGAL* family of *Gossypium hirsutum* was analyzed under different abiotic stresses and the results were visualized in the form of heat maps (Chen et al., 2020). According to the results, under cold stress, *GhAGAL4* and *GhAGAL11*, *GhAGAL2*, *GhAGAL15*, *GhAGAL6*, *GhAGAL3*, *GhAGAL9*, *GhAGAL13*, *GhAGAL5*, *GhAGAL12*, and *GhAGAL11* were upregulated whereas *GhAGAL1*, *GhAGAL2*, *GhAGAL3*, *GhAGAL8*, and *GhAGAL11* were downregulated. Under heat stress, *GhAGAL2*, *GhAGAL3*, *GhAGAL5*, *GhAGAL8*, *GhAGAL9*, *GhAGAL12*, *GhAGAL11*, and *GhAGAL13* were upregulated. *GhAGAL1*, *GhAGAL5*, *GhAGAL11*, and *GhAGAL13* were downregulated. Under PEG stress, the upregulated expressions of *GhAGAL8*, *GhAGAL2*, *GhAGAL3, GhAGAL9*, *GhAGAL11*, and *GhAGAL1* were downregulated. Under NaCl stress, *GhAGAL2*, *GhAGAL3*, *GhAGAL5*, *GhAGAL6*, *GhAGAL9*, *GhAGAL12*, *GhAGAL11*, and *GhAGAL1* were upregulated, whereas *GhAGAL1*, *GhAGAL3*, *GhAGAL11*, *GhAGAL12*, and *GhAGAL13* were downregulated.

### 3.5 Interaction network of GhAGAL proteins

**Figure 5:**
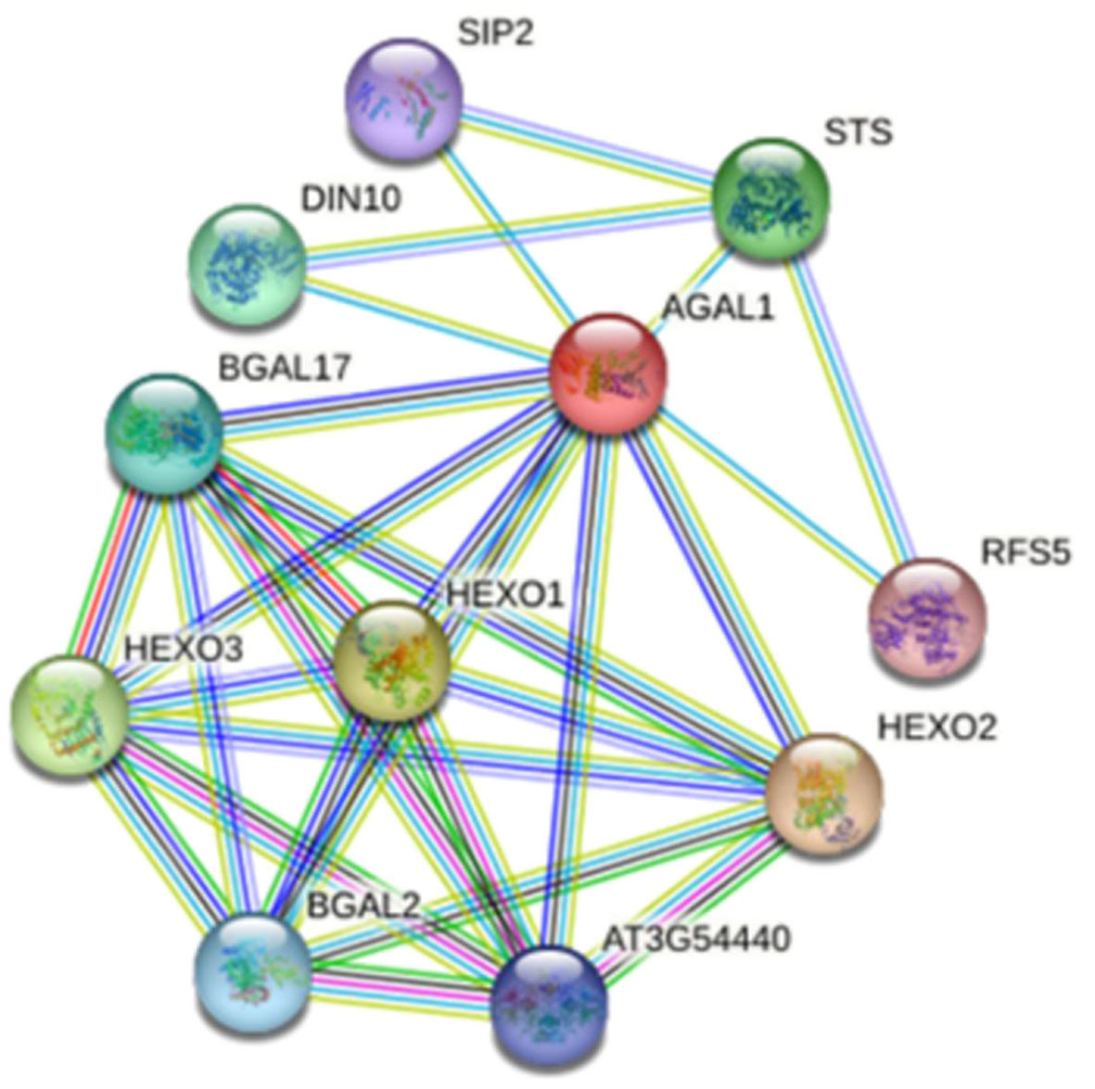
Interaction network of *AGAL* protein.

A protein interaction network map of GhAGAL was constructed based on the protein sequence of GhAGAL by adopting the online STRING database (https://string-db.org/) (Yu et al., 2009). In the predicted network, 10 proteins were predicted to interact with GhAGAL, including DIN10, STS, SIP2, RFS5, BGAL17, HEXO1, HEXO3, HEXO2, and BGAL2, (**Figure 5**). Afterward, the CottonFGD database was searched to identify the related genes that interact with GhAGAL. Among them, RFS5 interacted with GhAGAL and played a crucial role in mitigating salt stress in cotton. RFS5 not only helps scavenge free radicals but also enhances salt tolerance in cotton (Cui et al., 2021a). In this study, GhAGAL was identified as the decomposition gene of RFS5. Consequently, it was speculated that there might be a relationship between GhAGAL and RFS5.

### 3.6 Tissue-specific expression pattern of the *GhAGALs* gene and RT-qPCR analysis

**Figure 6:**
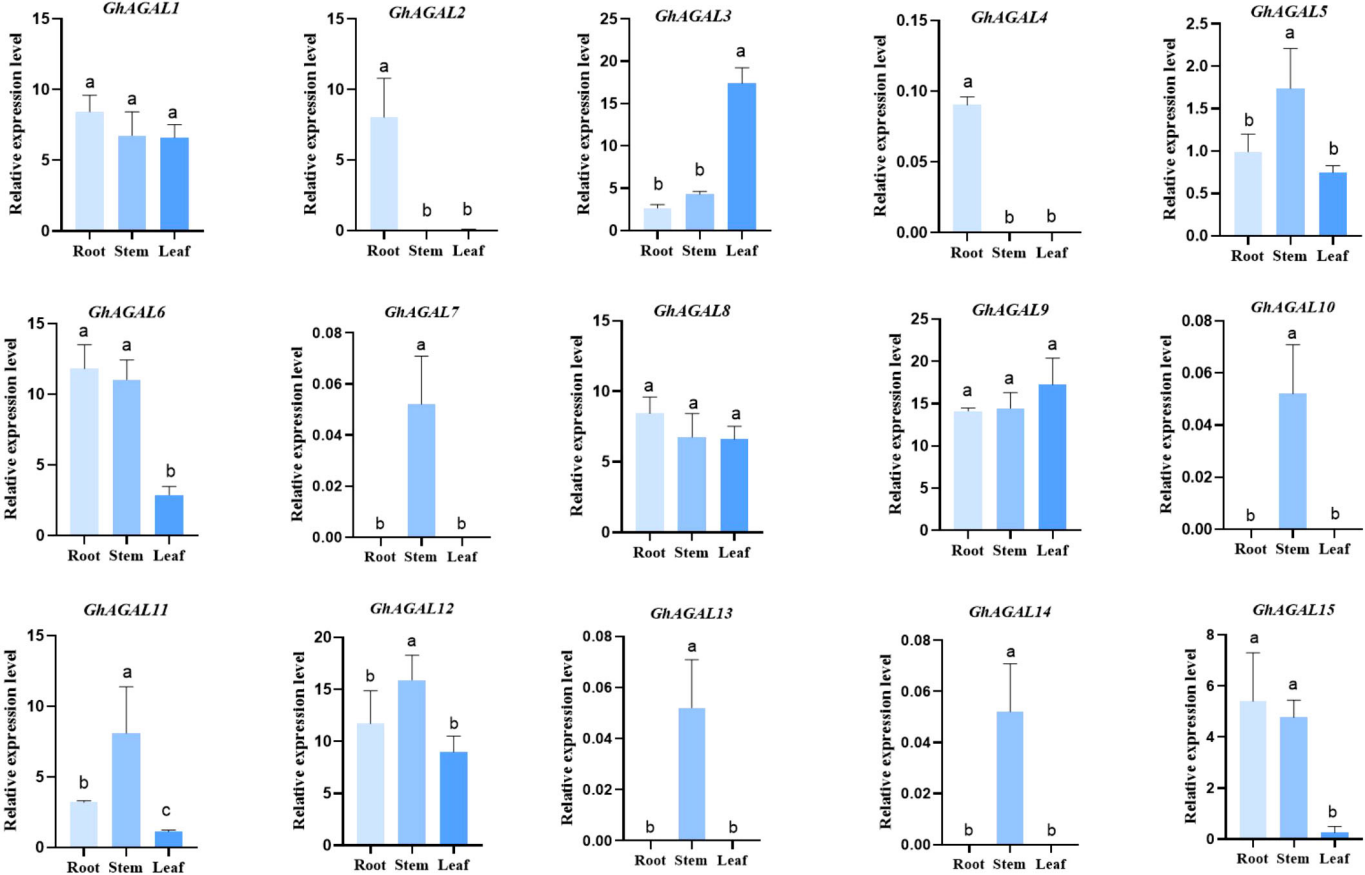
Specific expression of *AGAL* family genes in roots, stems, and leaves. Signifcance level α;=0.05: The resulting values are expressed in relative units. The error bar in the figure is the standard deviation (SD) of the three biological replicates in each treatment group.

To understand the expression of *AGAL* family genes in *Gossypium hirsutum*, the expression levels of 15 *AGAL* genes were analyzed in different tissues (roots, leaves, and stems) of *Gossypium hirsutum* (**Figure 6**). As a result, various genes exhibited significant differential expression within the same tissue, meanwhile the same also gene showed significant differential expression across different tissues. The expression pattern maps were created through clustering and analyzing differences in gene expression from a holistic perspective. These maps were divided into three categories, namely, high expression, no expression, and low expression. It was obviously observed from the graph that the relative expressions of *GhAGAL3* and *GhAGAL9* were the highest in leaves, whereas those of *GhAGAL5*, *GhAGAL11*, and *GhAGAL12* were the highest. The relative expression levels of *GhAGAL1*, *GhAGAL6*, and *GhAGAL8* were the highest in roots, where the relative expressions of *GhAGAL2*, *GhAGAL4*, *GhAGAL14*, and *GhAGAL15* were expressed only in roots and not in stems and leaves. *GhAGAL7* and *GhAGAL13* are expressed in stems, but not in roots and leaves.

**Figure 7:**
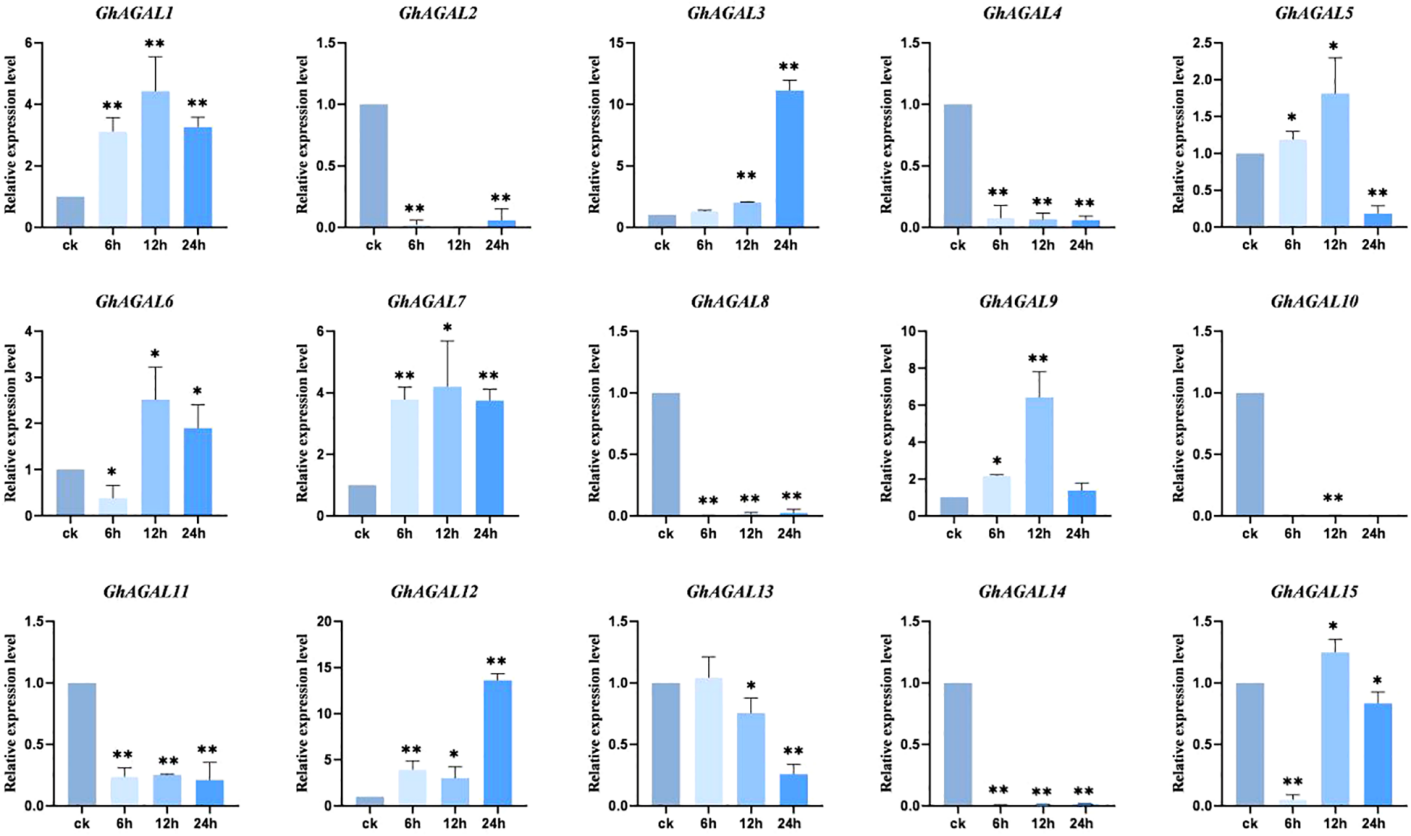
Expression of *AGAL* family genes in leaves at 0 h, 6 h, 12 h, and 24 h under NaCl stress. *0.01 < p < 0.05, **p < 0.01; the resulting mean values are presented as relative units.

To explore the effects of *AGAL* family genes on the plant tissue of *Gossypium hirsutum.* Quantitative reverse transcription-polymerase chain reaction (RT-qPCR) analysis was conducted, and a histogram was created to visualize the expression of 15 *AGAL* family genes in *Gossypium hirsutum* leaves under NaCl stress at 0 h (CK), 6 h, 12 h, and 24 h. As clearly observed from **Figure 7**, the expression level of *GhAGAL1* increased at 6 h and 12 h but decreased at 24 h compared with the control (CK). *GhAGAL2* expression decreased at 6 h, 12 h, and 24 h relative to the control group (CK), whereas *GhAGAL3* expression continued to increase at 0 h, 6 h, 12 h, and 24 h, and *GhAGAL4* expression decreased at 6 h, 12 h, and 24 h. The expression of *GhAGAL5* increased at 6 h and 12 h but decreased significantly at 24 h. *GhAGAL6* expression decreased at 6 h and then increased rapidly at 12 h compared with CK but decreased again at 24 h. *GhAGAL7* expression increased at 6 h, 12 h, and 24 h but decreased again at 24 h after increasing at 12 h. *GhAGAL8* expression decreased at 24 h compared with CK and increased at 0 h, 6 h, 12 h, and 24 h relative to CK. In addition, the expression of *GhAGAL8* decreased compared with the control (CK). *GhAGAL12* expression initially increased at 6 h and then slightly decreased at 12 h and later rapidly increased at 24 h compared with the control. *GhAGAL13* expression did not show any significant change at 6 h compared with the control but then continued to decrease at 12 h and 24 h. *GhAGAL14* expression decreased at 6 h, 12 h, and 24 h relative to the control, whereas *GhAGAL15* expression was also decreased at 6 h, 12 h, and 24 h compared with the control. The expression of *GhAGAL15* decreased significantly at 6 h, 12 h, and 24 h. It decreased significantly at 6 h compared with CK and then increased significantly at 12 h but decreased again at 24 h. *GhAGAL3* expression continued to rise with time under NaCl stress (**Figure 7**). Therefore, it was screened for functional verification by combining the tissue specificity of the *GhAGAL* family and conducting RT-qPCR analysis in leaves.

### 3.7 Phenotype of cotton plants with *GhAGAL3* gene silencing by VIGS under NaCl stress

**Figure 8:**
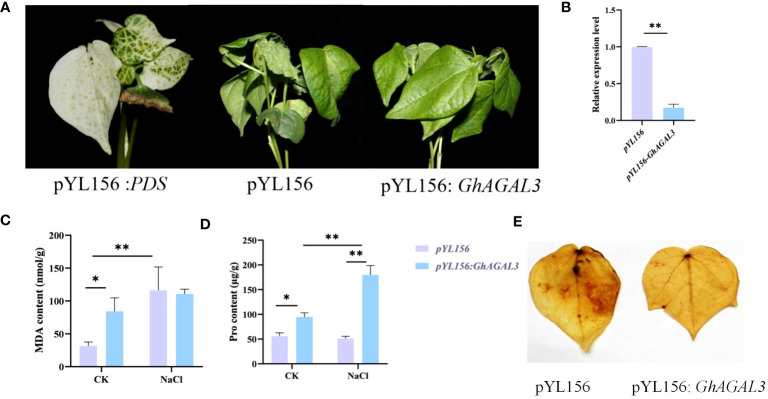
Phenotype of cotton leaves after virus infection and expression of *GhAGAL3* under NaCl stress. **(A)** Phenotype of cotton after *GhAGAL3* gene silencing under NaCl stress. pYL 156: PDS as a positive control, pYL156 was an empty vector as control, and pYL156 *GhAGAL3* was the *GhAGAL3*-silenced lines. **(B)** Relative expression level of *GhAGAL3* under NaCl stress. **(C)** MDA content of empty control and VIGS plants under normal growth and NaCl stress. **(D)** Pro content of empty control and VIGS plants under normal growth and NaCl stress. **(E)** DAB staining. *0.01 < p < 0.05, **p < 0.01; the resulting mean values are presented as relative units.

To validate the above result, the genetic virus-mediated gene silencing technique was performed on the *GhAGAL3* gene (**Figure 8**). When the cotton reached the three-leaf stage, PDS plants exhibited albinism. Then, plants injected with the pYL156 carrier and pYL156:*GhAGAL3* were transferred to a tripod and further treated with 100 mM NaCl. As a result, the plant phenotype of pYL156 was more wilted than that of pYL156: *GhAGAL3* at 36 h. The expression level of the plants was analyzed using RT-qPCR. The results showed that the expression level of *GhAGAL3* plants was lower than that of pYL156 carriers, which further indicated the successful gene silencing. At the same time, the MDA content of *GhAGAL3* gene-silenced plants decreased slightly after salt stress, whereas the Pro content increased significantly. In contrast, the MDA content of pYL156: *GhAGAL3* was higher than that of pYL156 in the case of CK treatment. However, under NaCl stress, there was a slight downward trend in the MDA content of pYL156: *GhAGAL3* compared with that of pYL156. This suggests that the degree of membrane lipolysis damage was also reduced, resulting in lesser damage to the plants. As revealed by DAB staining results, the leaves of pYL156 plants under NaCl stress were darker than those of *GhAGAL3* plants. Based on the above results, after *GhAGAL3* silencing, cotton maintained normal growth by regulating the contents of osmotic substances such as proline. Additionally, cotton scavenged ROS by improving the activities of antioxidant enzymes, thus enabling cotton to resist NaCl stress.

### 3.8 Changes of raffinose content under NaCl stress

**Figure 9:**
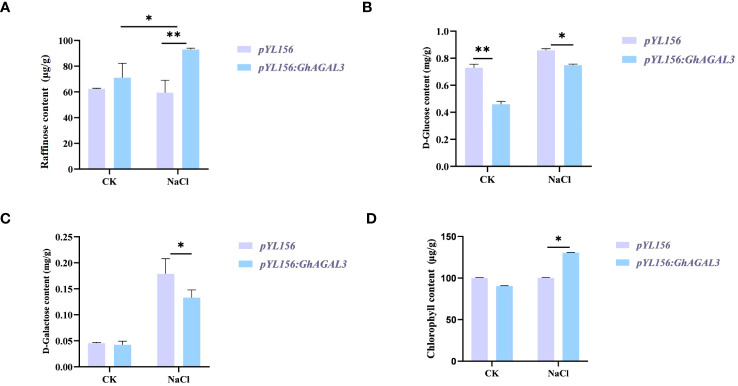
Determination of raffinose, D-glucose, D-galactose, and chlorophyll content after *GhAGAL3* silencing. **(A)** Raffinose content of empty control and VIGS plants under normal growth and NaCl stress. **(B)** D-glucose content of empty control and VIGS plants under normal growth and NaCl stress. **(C)** D-galactose content of empty control and VIGS plants under normal growth and NaCl stress. **(D)** content of empty control and VIGS plants under normal growth and NaCl stress. *0.01 < p < 0.05, **p < 0.01; the resulting mean values were presented as relative units.

To further investigate the potential relationship between raffinose and salt tolerance in cotton, the raffinose content was measured in both control and salt-treated plants before and after treatment (**Figure 9**). According to our results, the content of raffinose increased after silencing *GhAGAL3*. Meanwhile, the levels of D-glucose and D-galactose, which are the downstream metabolic substances controlled by *GhAGAL3*, were determined, respectively. As a result, the levels of D-glucose and D-galactose decreased under salt stress. the chlorophyll content of *GhAGAL3* plant increased significantly compared with that of the control plant pYL156. These results indicated that salt stress was alleviated by increasing the raffinose content.

## 4 Discussion

Due to global climate change, the abiotic environment has become one of the most crucial factors influencing plant growth and development. Salt stress, in particular, has a direct impact on crop growth, development, and yield (Vaughan et al., 2018). AGAL is a hydrolyzing enzyme related to raffinose, which has an irreplaceable role in plant growth and development, as well as in response to adversity (Tsaniklidis et al., 2016). Studies on the *AGAL* gene family have revealed that AGAL exists not only in cotton but also in other evolutionary branches of species, including *Arabidopsis thaliana*, *Oryza sativa*, *Populus trichocarpa*, *Vitis vinifera*, and *Zea mays*. To be specific, 15 AGAL genes were identified in *Gossypium hirsutum*, 15 in *Gossypium barbadense*, 7 in *Gossypium raimondii*, 7 in *Gossypium arboreum*, 9 in *Oryza sativa*, 7 in *Vitis vinifera*, 18 in *Zea mays*, 22 in *Populus trichocarpa*, and 4 in *Arabidopsis thaliana* (Chrost et al., 2007). There were 44 *AGAL* genes identified in the four major cotton species, with the same number being identified in *Vitis vinifera*, *Gossypium arboreum*, and *Gossypium raimondii*. Therefore, it was speculated that amplification might occur during the evolutionary process. Although less studied in plant *AGAL*, four members have been identified in *Arabidopsis thaliana*, but none of them has been reported in cotton as of now. At the same time, the constructed phylogenetic tree showed that cotton was closely related to *Zea mays* and distantly related to *Vitis vinifera*. In addition, *AGAL* genes tended to be conserved throughout the cotton genome. *AGAL* plays an important role in plant growth, development, and response to adversity stress (Zhou et al., 2012; Han et al., 2015).

From the perspectives of chromosome localization and motif structure analysis, it was found that the gene structure of the *AGAL* family was highly conserved. In addition, the structure of the *GhAGAL* gene family members was highly consistent with that of the *GbAGAL* gene family members both of which evolve from diploid to tetraploid.

The expression of *AGAL* genes is regulated by various environmental factors, including light, phytohormones, and adversity stress. The present study revealed the patterns of response to salt, PEG, cold, and heat stresses, as well as the tissue-specific expression of the *AGAL* gene family. Notably, the *cis*-acting element has an important function when plants are subjected to abiotic stresses(Yadav et al., 2011). According to our results, *cis*-acting elements responded and generated excitons to regulate gene expression. A large number of hormone response elements, such as salicylic acid, jasmonic acid, and abscisic acid, are present in the promoter of *GhAGALs*. In this study, corresponding *cis*-acting elements of salicylic acid, jasmonic acid, abscisic acid, and other plant hormones exerted a crucial effect plant adaptation to abiotic stresses. When cotton is exposed to abiotic stress, it undergoes a variety of physiological and biochemical responses to mitigate the adverse effects of the stress (Zhang et al., 2022). Abiotic stresses including drought, cold, heat, and salt can reduce cotton yield. This study provided the first comprehensive analysis of the *AGAL* gene family in four cotton species. Finally, to verify the function of the *GhAGAL3* gene, we silenced it through the VIGS experiment. The results showed that silencing *GhAGAL3* reduced the salt tolerance of cotton.

Studies have reported that the metabolism of RFOs is a complex regulatory network in plants, and numerous associated enzymes are involved in the accumulation of RFOs (Sui et al., 2012). The first step in the catabolism of raffinose is the hydrolysis mediated by α-Gal, which produces sucrose and galactose (Peng et al., 2014). RFOs are distributed in plants and exert a protective effect in response to a wide range of abiotic stresses (Sonali et al., 2015). Overexpression of the *α-Gal* gene decreases the level of raffinose, which in turn reduces the cold tolerance of plants (Salvi et al., 2022). In the meantime, low temperature induced an increase in fructose content and a decrease in sucrose content within cucumber stems (Dos Santos et al., 2011). Moreover, the alkaline *α-Gal* activity of stem samples was lower than that of control samples after low temperature stress. Thus, *α-Gal* plays an important role in inhibiting glucose metabolism.

**Figure 10:**
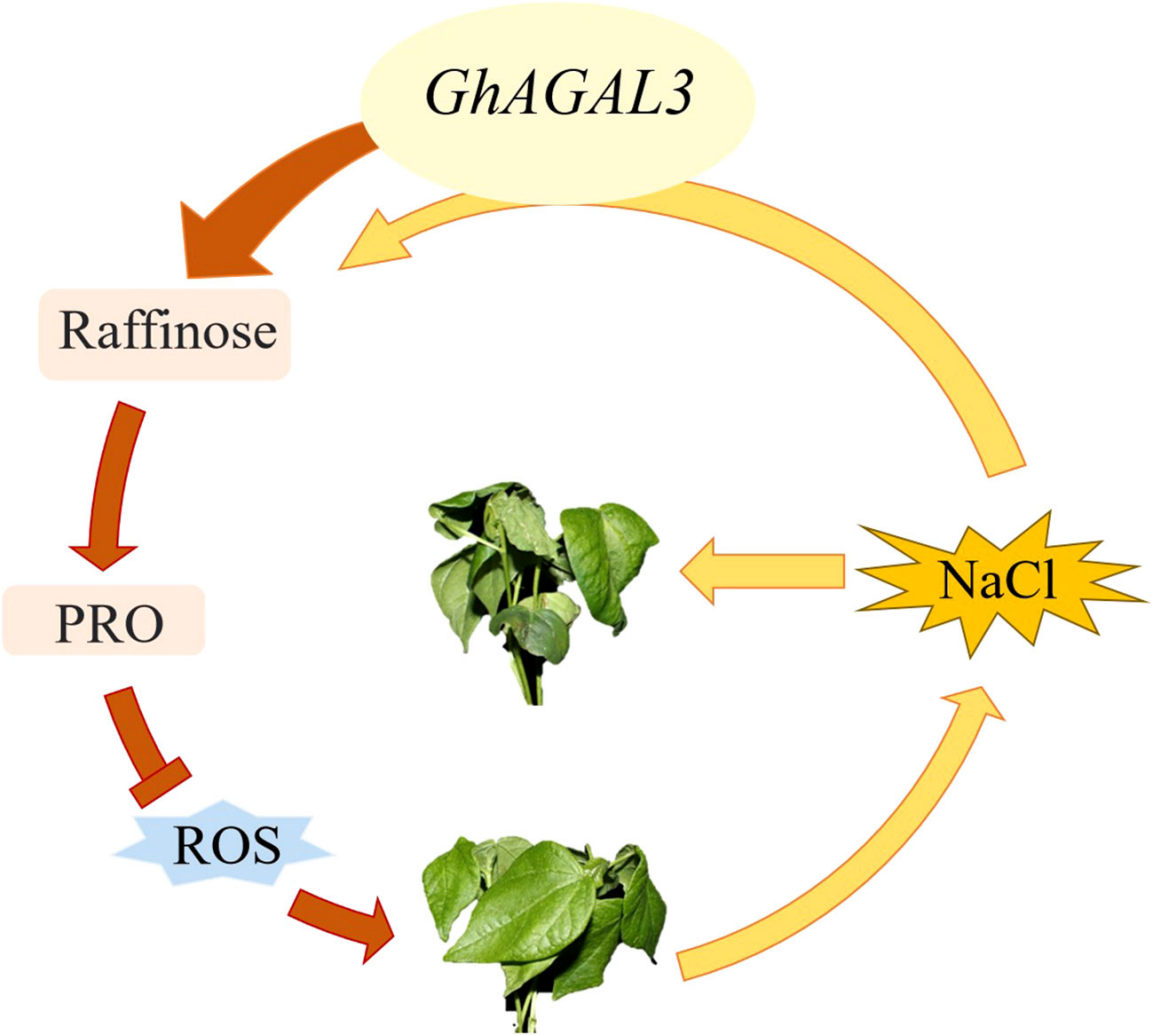
Mechanism model of *GhAGAL3* in regulating cotton response to NaCl stress.

According to the expression patterns of *AGAL* gene in different tissues under NaCl stress and under different abiotic stresses, *GhAGAL3* was selected for a functional verification study. After silencing *GhAGAL3* gene, the wilting degree of seedlings decreased relative to the negative control. It was inferred that *GhAGAL3* might play an important role in responding to NaCl stress (**Figure 10**). Under NaCl stress, the expression of *GhAGAL3* decreased and the raffinose content increased. Additionally, the proline content of *GhAGAL3* significantly increased. Typically, the proline content in plants reflects the extent of their resistance to stress. Plants accumulate proline under stressful conditions. The higher the proline content, the stronger the resistance (Xie et al., 2011). When plants accumulate an excessive amount of reactive oxygen species, lipid peroxidation occurs, leading to the production of free radicals. Under such circumstances, plants are affected by oxidative stress, and as a result, cells cannot function normally. Lipid peroxidation is the most serious injury process in an organic body. As revealed by the DAB staining in this study, the leaves of pYL156 plants treated with NaCl were darker than those of *GhAGAL3*, which might be ascribed oxidative stress. Brown spots were clearly visible on cotton leaves under stress, and the color of veins was darker. This suggested the significant buildup deepening, indicating the accumulation of ROS in cotton leaves after the stress treatment (Montillet et al., 2005). Therefore, it was concluded that increasing the raffinose content in cotton seedlings effectively eliminated hydroxyl free radicals and reduced the salt stress-induced damage to cotton seedlings. To enhance the salt stress tolerance of cotton to salt stress, raffinose is used as an osmotic regulator in previous studies to alleviate salt stress and eliminate hydroxyl radicals from the body. ROS production in plants is reduced under stress. (Nishizawa et al., 2019). In some studies, the mechanism by which *CsGolS4* enhances drought resistance is illustrated, and whether *CsGolS4* improves oxidative damage caused by low temperature and drought stresses is examined by measuring the activities of reactive oxygen enzymes (Ma et al., 2021). Various environmental stresses induce the accumulation of ROS whereas an excessive amount of ROS can cause cell damage, including lipid peroxidation damage (Van den Ende and Valluru, 2008). Collectively, these studies suggest that RFOs play an important role in enhancing stress resistance in plants.

## 5 Conclusion

According to the findings in this study, raffinose played a vital role in salt stress. The phenotype and raffinose content of cotton leaves under salt stress were investigated in this study. *AGAL* genes were discovered in cotton, and 7, 15, 15, and 7 *AGAL* genes were found in *Gossypium arboreum*, *Gossypium barbadense*, *Gossypium hirsutum*, and *Gossypium raimondii*, respectively. The AGAL genes were divided into three branches based on the phylogenetic tree, gene structure, and motifs. The *AGAL* family was engaged in a variety of abiotic stressors. Raffinose played a crucial role in salt stress. Therefore, silencing *GhAGAL3* resulted in a more favorable phenotype under NaCl stress. Results in this study can lay a theoretical foundation for further research on the link between *GhAGAL3* and NaCl stress.

## Data availability statement

The original contributions presented in the study are included in the article/**Supplementary Material**. Further inquiries can be directed to the corresponding authors.

## Author contributions

WC, YC, YH, LZ, and RC: Designed the experiments, methodology, experiment, analysis of data, writing-original draft preparation, writing-review and editing. XYL: Methodology, experiment. HH: Methodology. YF and YZ: Experiment. XF, KN, and TJ: Experiment. YM and ML: Experiment. MH and YL: Experiment. XKL, XC, and DW: Methodology. LZ, LG, JW, and SW: Methodology. QC: Writing-review and editing. WY: Conceived and designed the experiments, supervision. All authors contributed to the article and approved the submitted version.
