# Supplementary material for: Raffinose degradation-related gene GhAGAL3 was screened out responding to salinity stress through expression patterns of GhAGALs family genes
Source: Front Plant Sci. 2023 Dec 19;14:1246677. doi: 10.3389/fpls.2023.1246677 (PMC10773686; doi:10.3389/fpls.2023.1246677)
Supplement: Supplementary file 1 [file Table_1.docx]

Supplementary Table S1 Primer pairs for fluorescence quantification of *AGAL* gene family

| Gene ID | Primer sequence |
| --- | --- |
| *GhAGAL1* | F: TAGATTCCGGCAGACAGTGG |
|  | R: CTTCCAGCCTCTAGCTTGGT |
| *GhAGAL2* | F: TGGATGCAAGCGATCAACAG |
|  | R: TTTGGCCTACGACCTCAGTT |
| *GhAGAL3* | F: TGGTTCAAGCCAGAGACCTT |
|  | R: TTGCATGAATGCGGTCTCAA |
| *GhAGAL4* | F:GTAATGAGTACCGCCGATGC |
|  | R:CAGCCCAACAGTCATCAAGG |
| *GhAGAL5* | F: TGGACCTGGAGGATGGAATG |
|  | R: GGCCTTCATCAAAGCCCAAA |
| *GhAGAL6* | F: TGATCCGGACATGCTTGAGA |
|  | R:CTTATGTCGCAGCCGAGAAG |
| *GhAGAL7* | F: CTATCGGTCCTCGGCTTCTT |
|  | R: TAGGTGCTAGCATGTTCTGGTT |
| *GhAGAL8* | F: TGGATGCAAGCGATCAACAG |
|  | R: TTTGGCCTACGACCTCAGTT |
| *GhAGAL9* | F:CTGTGTTGTGGCGGCATTAG |
|  | R: CAACCCATGGGAGGAGTCAT |
| *GhAGAL10* | F:CTGTGTTGTGGCGGCATTAG |
|  | R: CAACCCATGGGAGGAGTCAT |
| *GhAGAL11* | F: TGTTCGATCTGCGAGCAAAG |
|  | R: CTAGGCCTCCATTGGATCGT |
| *GhAGAL12* | F: CCAATGCGTGACGCTCTAAA |
|  | R:TACGCCAGCTATTTCCGACT |
| *GhAGAL13* | F: TGACGGGAATAGCGGATCAA |
|  | R: GTTGCCTACTTCCAGCATGT |
| *GhAGAL14* | F: AGCTGGAGGACAACTTGTGA |
|  | R: GCATGTCCGGATCATTCCAA |
| *GhAGAL15* | F: AGGATGCACGGTGATGAAGA |
|  | R:AACGCGGTTACAGAATAGCG |
